# The Reactive Extrusion Cationization of Starches as a Novel Physicochemical Surface Modification Method

**DOI:** 10.1002/bip.70093

**Published:** 2026-03-31

**Authors:** Maria C. Posada‐Vélez, Beatriz M. Millán‐Malo, Oscar Y. Barrón‐García, Marcela Gaytán‐Martínez, Germán Buitrón

**Affiliations:** ^1^ Universidad Nacional Autónoma de México Posgrado en Ciencia e Ingeniería de Materiales, Centro de Física Aplicada y Tecnología Avanzada, Campus Juriquilla Queretaro Mexico; ^2^ Departamento de Nanotecnología, Centro de Física Aplica y Tecnología Avanzada Universidad Nacional Autónoma de México Queretaro Mexico; ^3^ Programa de Posgrado en Alimentos del Centro de la República (PROPAC) Universidad Autónoma de Querétaro, Centro Universitario Queretaro Mexico; ^4^ Divisón Industrial Universidad Tecnológica de Querétaro Queretaro Mexico; ^5^ LIPATA Universidad Nacional Autónoma de México, Instituto de Ingeniería, Unidad Académica Juriquilla Queretaro Mexico

**Keywords:** cationization, chinchayote starch or chayote root ( *Sechium edule* (Jacq.) Sw.) starch, corn starch ( *Zea mays* everta), glycidyltrimethylammonium chloride (GTAC), reactive extrusion (REX), sustainable processing

## Abstract

The modification of starches by cationization is a fundamental physicochemical process aimed at improving their physicochemical properties and expanding their industrial applications. Traditionally, this modification is associated with long duration, high energy consumption, and waste generation. This article proposes a method based on reactive extrusion (REX) as a sustainable alternative for modifying corn ( *Zea mays* everta) and chayote roots ( *Sechium edule* ) or chinchayote starches. A single‐screw extruder was utilized to assess the effects of temperature on the degree of substitution (DS) and the functional and structural properties of the modified starches. Glycyltrimethylammonium chloride (GTAC) was used as a cationizing agent in both methods, REX and conventional cationization (CT), at a concentration of 3%. The results indicate that extrusion can produce starches with a DS equivalent to that obtained by CT in the case of corn starch (0.21–0.23). Rheometry shows a decrease in the viscosity peaks due to the pre‐gelatinization process. Calorimetry showed a decrease in enthalpy and an increase in tractability temperature for the REX‐modified starches due to the temperature and shear to which they were subjected. The spectroscopic technique showed the incorporation of GTAC into the starch structure. The results of physicochemical characterization show that the REX is identified as a viable alternative to CT, offering a faster, more energy‐efficient, and environmentally friendly process. The effectiveness of REX in altering the physicochemical properties of starch suggests its potential for innovative industrial applications, such as water treatment or the production of biodegradable materials.

## Graphical abstract

Native starches from different botanical sources were cationized by reactive extrusion using 3% glycidyltrimethylammonium chloride (GTAC). The process, performed under controlled thermal zones, promoted the incorporation of quaternary ammonium groups onto the starch backbone. This physicochemical surface modification enhances the cationic character of starch, improving its functional performance and expanding its potential as a sustainable, bio‐based coagulant and flocculant.

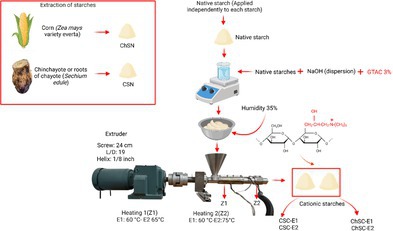

## 1 Introduction

Starch cationization is a chemical modification process that introduces positively charged functional groups—typically quaternary ammonium moieties—into the starch macromolecule, conferring cationic character and enhancing its affinity for negatively charged substrates [1]. Although the properties of native starches vary with their botanical source, their applications are often limited by characteristic properties such as low solubility, low shear strength, poor thermal stability, and a high tendency to retrograde [2, 3]. Cationization addresses these limitations by substituting hydroxyl groups with cationic substituents, improving water binding capacity, viscosity stability, and electrostatic interactions with anionic surfaces. As a result, cationized starches are widely used in sectors such as papermaking, textiles, adhesives, personal care products, and, more recently, wastewater treatment, where they serve as efficient coagulants and flocculants capable of removing colloidal particles and pathogenic microorganisms such as *Escherichia coli* [4, 5, 6].

Conventional cationization (CT) is typically performed in aqueous alkaline media using reagents such as 2,3‐epoxypropyltrimethylammonium chloride or 3‐chloro‐2‐hydroxypropyltrimethylammonium chloride (CHPTAC), with glycyltrimethylammonium chloride (GTAC) as a suitable alternative. The process begins with alkalization using NaOH to activate hydroxyl groups, followed by nucleophilic substitution under controlled temperature and stirring for extended periods (typically more than 6 h). Although effective, CT has several drawbacks: long reaction times, high energy and water consumption, excess reagent use, the generation of saline by‐products and chemical waste, and multistage operations involving neutralization, washing, and recovery [7, 8, 9]. These limitations hinder industrial scalability and conflict with the increasing demand for greener, more sustainable manufacturing processes [10].

In response to these challenges, reactive extrusion (REX) has emerged as a promising thermomechanical platform for the continuous, solvent‐free, or solvent‐minimized chemical modification of biopolymers [11, 12]. Extrusion combines thermal energy, high shear, and pressure in a single continuous operation, inducing significant structural transformations in starch, including granular disruption, loss of crystallinity, molecular depolymerization, and partial or complete gelatinization [13]. These thermomechanical effects increase the accessibility of reactive sites within the amorphous and crystalline domains, facilitating chemical reactions that would otherwise proceed slowly or require harsh conditions in batch processes. When coupled with cationizing agents such as GTAC, reactive extrusion enables one‐step, continuous cationization with significantly reduced residence times (minutes versus hours), lower reagent stoichiometry, and minimal effluent generation [14, 15].

Recent studies have demonstrated the versatility of reactive extrusion for starch functionalization, Marim et al. [16] demonstrated that a single screw can effectively function as a thermomechanical reactor for esterifying cassava starch with organic acids. This process enables rapid, scalable, and energy‐efficient hydrogel production. Wei et al. [17] used reactive extrusion as a continuous thermomechanical platform to induce cooperative crosslinking of starch with citric acid and Zn^2+^ ions; the resulting starch gels showed significantly improved mechanical performance, including a substantially increased elastic modulus and enhanced structural stability. Similar results were reported by Wei et al. [18], who demonstrated that combining a safe cross‐linking agent, citric acid, with Ca^2+^ cations in a continuous extrusion process enables the production of modified starch capable of forming gels with superior and tunable mechanical properties. This one‐step extrusion strategy promotes more efficient cross‐linking at relatively low temperatures compared to conventional methods, thereby improving energy efficiency and enhancing the uniformity and stability of the resulting cross‐linked network. These studies underscore the potential of REX as a scalable, energy‐efficient, and environmentally benign alternative to conventional batch processing.

Nevertheless, the application of reactive extrusion to underutilized starch sources– particularly those from tubers– remains largely unexplored. Among these, chinchayote or chayote root ( *S. edule* (Jacq.) Sw.) is an intriguing candidate. Cultivated extensively in Mesoamerica, this crop yields starch with distinctive physicochemical characteristics, including small granule size, high amylopectin content, and the presence of phosphate esters [16]. Preliminary studies by Morales‐Santiago et al. [19] investigated the chemical modification of chayote root starch with lauric acid and acetic anhydride, reporting improved thermal stability and water retention capacity. However, to date, no study has addressed the cationization of chinchayote either by conventional means or by reactive extrusion—leaving a significant gap in knowledge regarding its functionalization potential and structure–property relationships.

Concurrently, maize ( *Z. mays* variety everta), commonly known as popcorn maize, is a significant economic and cultural starch source in Mexico. Its hard endosperm provides unique structural properties, including high expansion volume and resistance to mechanical damage. Previous studies have examined the conventional cationization of corn starch using quaternary ammonium reagents under alkaline conditions, showing that the degree of substitution (DS) increases with reagent concentration and reaction time [20, 21]. However, comparative assessments between conventional and extrusion‐based cationization of this botanical variety are still lacking.

It is important to clarify that this study does not aim to evaluate the physical effects of extrusion as an independent treatment. Instead, extrusion is used as a reactive processing technology to facilitate the covalent attachment of cationic groups. Consequently, non‐reactive extrusion controls were not included in the experimental scope. Nonetheless, it is recognized that certain properties—particularly pasting behavior and thermal transitions—are inherently influenced by the applied thermomechanical conditions. Therefore, selected studies on physically extruded starches are cited solely to contextualize the observed trends and support the interpretation of processing‐induced effects, not to establish direct comparisons with non‐reactive controls.

This study presents the first systematic comparison of conventional and reactive extrusion cationization applied to two botanically distinct starch sources: corn ( *Z. mays var. everta* ) and chayote ( *S. edule* ) root. The primary objectives are: (i) to evaluate the degree of substitution (DS) achieved by each method under a fixed reagent concentration (3% GTAC); (ii) to characterize the structural, morphological, thermal, and rheological changes induced by both modification routes; and (iii) to critically assess the feasibility of reactive extrusion as a faster, cleaner, and industrially scalable alternative to conventional cationization. By integrating a previously unexplored botanical source into the reactive extrusion framework, this work contributes to broadening the feedstock portfolio for sustainable starch modification and valorizes an underutilized agricultural resource. The findings are expected to inform future process optimization efforts and support the development of novel applications in water treatment, biodegradable materials, and bio‐based flocculants.

## 2 Materials and Methods

### 2.1 Sample Description

The corn from the popcorn variety ( *Z. mays* variety everta) was purchased at a local market in Querétaro. In contrast, the chinchayote or chayote ( *S. edule* ) roots were obtained from Chiapas, México. Glycyltrimethylammonium chloride (GTAC) was purchased from Sigma‐Aldrich Co, USA. All other materials or chemicals are of analytical grade and were used as received without further modification.

### 2.2 Starch Isolation

The methodology proposed by Pineda‐Gomez et al. [22] and Londoño‐Restrepo et al. [23] was applied to isolate starch from corn (CSN) and chinchayote (ChSN). For the corn starch samples, 5 kg of the material was wet milled for 60 s and then dried for an additional 30 s. The resulting mixture of water and starch was filtered through a 100 μm sieve. The samples were centrifuged at 4500 rpm for three cycles to separate the starch from other components (fat and proteins) in the liquid fraction. The paste was then dried in a conventional oven at a temperature of 40°C for 12 h. The resulting dry material was finely pulverized and sieved using a 60 mesh.

In ChSN, the roots were washed, peeled, and cut into small pieces of 2 × 2 × 1 cm, then kept in distilled water to prevent oxidation. The wet samples were processed for 30 s using a food processor (Black & Decker, Ergo 5). The solution was filtered twice through an 80 μm and a 200 μm sieve. The residue remaining in the sieve was sprayed again, and this process was repeated three times. Finally, the solution was poured into jars and stored at 4°C for 12 h to create a precipitated compact paste. First, the precipitate was dissolved and centrifuged at 4500 rpm for 10 min; then it was washed with a 1% bisulfite solution, and this procedure was performed three times. It was then dried in an oven at 40°C for 12 h. Finally, the obtained starch was ground and sieved through a #60 sieve.

### 2.3 Chemical Proximal Analysis

After extraction of CSN and ChSN starches different physicochemical characteristics were determined according to the AOAC 2000 Official Methods of Analysis for moisture (925.10), lipid (920.39), ash (945.46), and protein with the kjendahl method (990.03). Amylose content was determined by means of a colorimetric method based on the formation of an amylose‐iodine complex [24]. The carbohydrate content was calculated by the difference. Each analysis was performed in triplicate.

### 2.4 Starch Modification

To determine the yield of both methods, isolated starch was subjected to chemical and thermomechanical modification. In this case, the extruder was used as a bioreactor for the thermomechanical modification.

#### 2.4.1 Conventional Chemical Modification (CT)

The cationic starches derived from corn (CSC‐C) and chinchayote (ChSC‐C) were prepared following the method proposed by Abdo et al. [20]. First, 20 g of native corn starch was dispersed in a 1 mol/L NaOH solution and maintained at 40°C for 2 h with mechanical stirring. Next, 3 mL of GTAC was added to the complete dispersion of native starch, and the solution was again kept under constant mechanical stirring for 6 h, ensuring the pH was adjusted to 7. At the end of the cationization process, absolute ethanol was added dropwise to terminate the reaction and precipitate the cationic starch. No additional reaction time was applied during the ethanol addition step, as ethanol was used solely as a quenching and precipitation agent. Finally, the starch slurry was washed three times with an ethanol/H2O (70/30) to eliminate the unreacted GTAC and NaOH compounds. The resulting products were dried under vacuum at 40°C for 12 h. The dry sample was ground and sieved using a 60 US (250 mm) sieve.

#### 2.4.2 Reactive Extrusion Modification (REX)

A single‐screw extruder with two heating zones was utilized, developed by CICATA‐IPN Querétaro (Mexico) (patent MX/a/2007/016262). The screw has a diameter of 2.54 cm, an L/D ratio of 19, and a helix depth of 1/8 in. An outlet‐forming nozzle with a 5 mm opening was attached to the extruder. Two experiments were performed to test this method. In the first experiment, 20 g corn starch (CSC‐E1) and 20 g chinchayote starch (ChSC‐E1) were dispersed in a 1 mol/L NaOH solution, and 3% GTAC was added at a concentration of 3% (w/w) relative to the dry starch mass. The mixture was magnetically stirred for 30 min, allowed to rest for 90 min, and subsequently analyzed using a thermobalance to adjust the moisture content to 35%. The starch samples were extruded at 60°C in both heating zones and at a screw speed of 50 rpm. The cationized starch was processed under these conditions for a total residence time of 30 min, followed by drying in an oven at 40°C for 12 h. Subsequently, the dried material was ground and passed through a #60 mesh sieve.

**FIGURE 1:**
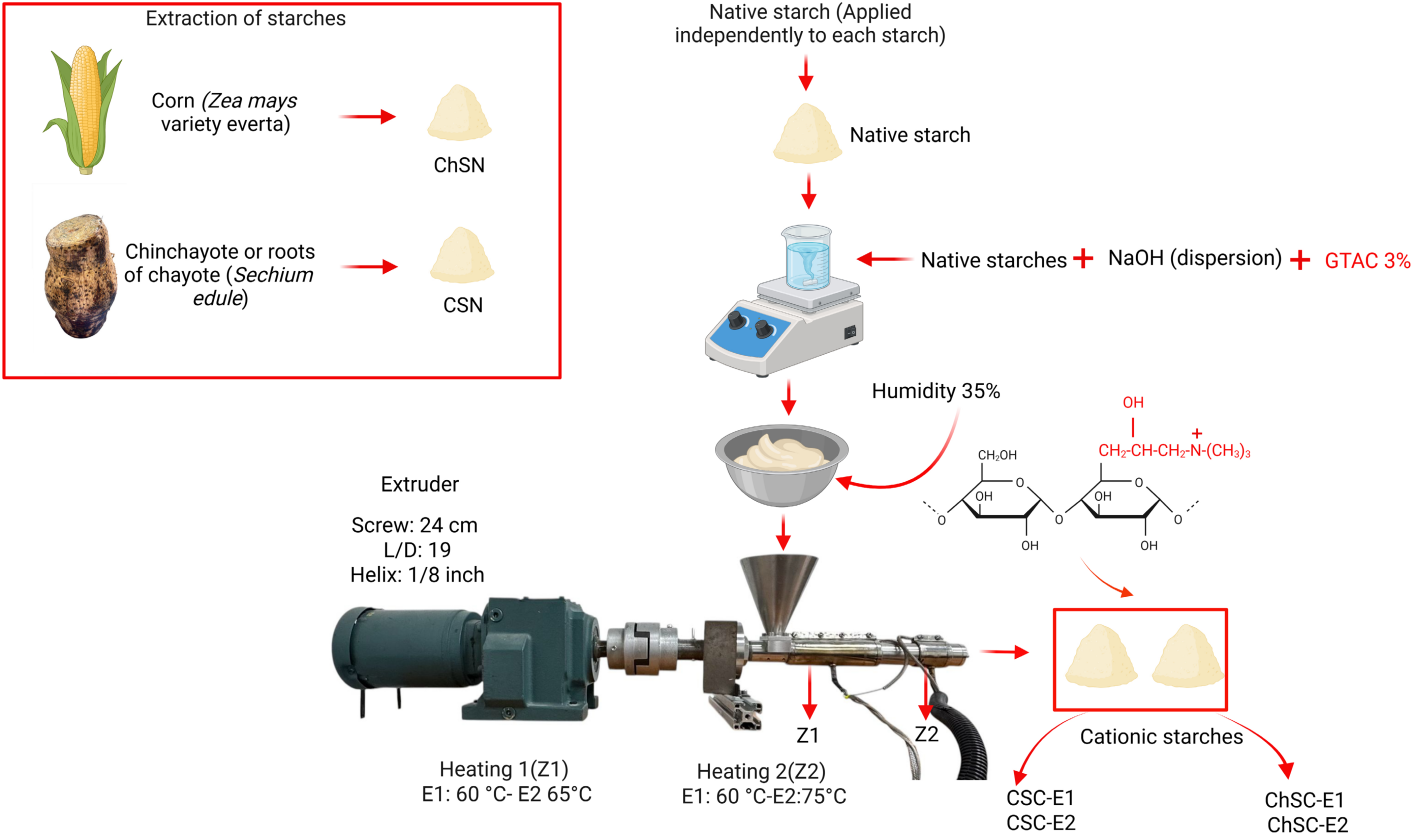
Reactive extrusion process of corn and chinchayote starches.

In the second experiment, corn starch (CSC‐E2) and chinchayote starch (ChSC‐E2) were modified under the same processing conditions but extruded at two different temperatures: 60°C in the first heating zone and 75°C in the second heating zone. These temperatures were selected based on preliminary experiments and considering the extrusion conditions reported by Cervantes‐Ramírez et al. [10], they used temperatures of 100°C and 85°C in their study. The overall extrusion and cationization process is illustrated in Figure 1.

### 2.5 Physicochemical Characterization

#### 2.5.1 Determination of the Degree of Substitution of the Modified Starches

The degree of substitution (DS) was determined by measuring the nitrogen content of the modified starches. The Kjeldahl method was employed to assess the nitrogen content of the modified starches [25]. Consequently, the DS can be calculated using the following equation:

$$\mathrm{DS}=\frac{162.1\times N\%}{14.01\times 100-151.64\times N\%}$$

In this context, N% represents the nitrogen content ratio. The atomic weight of nitrogen is 14.01, the molecular weight of an anhydrous glucose unit of starch is 162.14, and the molar number of substituted groups is 151.64. The test was made in triplicate.

#### 2.5.2 Scanning Electron Microscopy (SEM)

Scanning electron microscope (SEM) images of isolated starches (CSN and ChSN), starches modified by the conventional method (CSC‐C and ChSC‐C), and starches modified by the extrusion method (CSC‐E1, CSC‐E2, ChSC‐E1, and ChSC‐E2) were obtained using a JSM‐6060LV scanning electron microscope (JEOL Ltd., Japan). The starches were deposited on SEM aluminum stubs and coated with a cathodic gold/palladium (60:40) powder. Granule size analysis was performed by measuring the diameter of 100 starch granules from the SEM micrographs using ImageJ software (NIH, USA).

#### 2.5.3 X‐Ray Diffraction Powder Analysis

X‐ray diffraction (XRD) was utilized to analyze the native starches (CSN and ChSN), the modified starches created by the conventional method (CSC‐C and ChSC‐C), and the modified starches produced through the extrusion method (CSC‐E1, CSC‐E2, ChSC‐E1, and ChSC‐E2). X‐ray diffraction (XRD) patterns were recorded using an Ultima IV diffractometer (Rigaku, Tokyo, Japan) operating at 40 kV and 30 mA, with CuK_α_ radiation *λ* = 1.5406 Å. The measurements were taken from 4° to 60° on a 2θ scale, with an increment of 0.02° step size and a rate of 2 degrees per minute.

The relative crystallinity (RC) was calculated by the equation:

$$\mathrm{RC}\left(\%\right)=\frac{\mathrm{Ac}}{\mathrm{Ac}+\mathrm{Aa}}\times 100\%$$

where Ac and Aa represent crystalline and amorphous areas, respectively. They were calculated by selecting the diffraction angle (2θ = 10°–40°), baseline correction, and peak fitting.

#### 2.5.4 Differential Scanning Calorimetry

Thermal analysis of native starches (CSN and ChSN), starches modified by the conventional method (CSC‐C and ChSC‐C), and starches modified by the extrusion method (CSC‐E1, CSC‐E2, ChSC‐E1, and ChSC‐E2) was conducted using a DSC‐Q200 calorimeter (TA Instruments, New Castle, DE, USA) at a heating rate of 5°C/min from room temperature to 95°C under a nitrogen atmosphere (50 mL/min). Suspensions at a ratio of 2:12 of these starches were prepared in a sealed aluminum DSC capsule. This analysis was performed on two occasions.

#### 2.5.5 Pasting Properties

The pasting properties of native starches (CSN and ChSN), starches modified by the conventional method (CSC‐C and ChSC‐C), and starches modified by the extrusion method (CSC‐E1, CSC‐E2, ChSC‐E1, and ChSC‐E2) were determined using a rheometer (Anton Paar MCR‐102; Austria) equipped with a starch cell. Starch‐water suspensions were prepared with 3.0 g of starch (dry basis) and 18.0 mL of distilled water, yielding a starch concentration of 14.3% (w/w). In this test, each suspension was heated and subjected to shear forces as follows: First, the system temperature was maintained at a constant 50°C for 1 min, then heated to 90°C at a rate of 7.5°C/min, holding at 90°C for 5.3 min, cooling back to 50°C at −7.5°C/min, and stabilizing at 50°C for an additional minute. Measurements were conducted in triplicate.

#### 2.5.6 Fourier Transform Infrared Spectroscopy (FTIR)

The native starches (CSN and ChSN), starches modified by conventional methods (CSC‐C and ChSC‐C), and starches modified by the thermomechanical method (CSC‐E1, CSC‐E2, ChSC‐E1, and ChSC‐E2) were analyzed using a Perkin Elmer IR spectrometer (model Spectrum Two, Waltham‐USA) with ATR (attenuated total reflectance). The infrared spectra were obtained in the range of 600–4000 cm^−1^.

#### 2.5.7 Statistical Analysis

Statistical analyses were conducted using the ANOVA test, followed by a post hoc analysis with multiple comparisons using Tukey's test (*p* ≤ 0.05), and values represent the mean of three measurements (*n* = 3) ± standard deviation.

## 3 Results and Discussion

### 3.1 Chemical Proximal Analysis

Chemical proximal analysis was performed to determine the moisture, lipid, ash, protein, and carbohydrate contents of isolated CSN and ChSN starches. These values are shown in Table 1. While corn starch (CSN) has been extensively characterized in the literature, limited information is available for chinchayote starch (ChSN), making its characterization particularly relevant.

**TABLE 1:** Chemical proximal values of CSN and ChSN.

Analysis (%)	CSN	ChSN
Moisture	4.18 ± 0.01	4.00 ± 0.04
Ash	0.50 ± 0.26	0.69 ± 0.04
Protein	0.39 ± 0.06	3.04 ± 0.01
Lipid	6.61 ± 0.25	7.14 ± 0.11
Carbohydrate	88.30 ± 0.10	85.10 ± 0.07
Amylose	26.30 ± 0.19	19.38 ± 0.06
Amylopectin	73.70 ± 0.15	80.62 ± 0.06

The results indicate that ChSN exhibits higher protein, lipid, and amylopectin contents than CSN. Comparable values for moisture, protein, ash, and lipid contents have been previously reported for chinchayote starch [19]. The significant differences observed in moisture and lipid values can be attributed to compositional variations and the varieties studied [26].

Some authors reported similar results for ChSN [27, 28]. However, the results obtained for ChSN were compared with those reported for potato starches, given their shared classification as tubers [19]. Alvani et al. [29] reported a protein content of 0.33% for various potato varieties. Conversely, Yang et al. [30] documented a lower protein and ash content of 0.23% and 0.24%, respectively, for the yellow potato variety. Despite their common botanical origin, substantial disparities emerge among protein and fat content with ChSN. The protein content plays a fundamental role in water absorption, and the starch‐lipid resulting from the formation of inclusion complexes with amylose and amylopectin molecules, among other factors [31]. However, the values reported for each analysis are within the limits for isolated starches according to NMX F‐3821986.

### 3.2 Degree of Substitution (DS) of the Modified Starches

**FIGURE 2:**
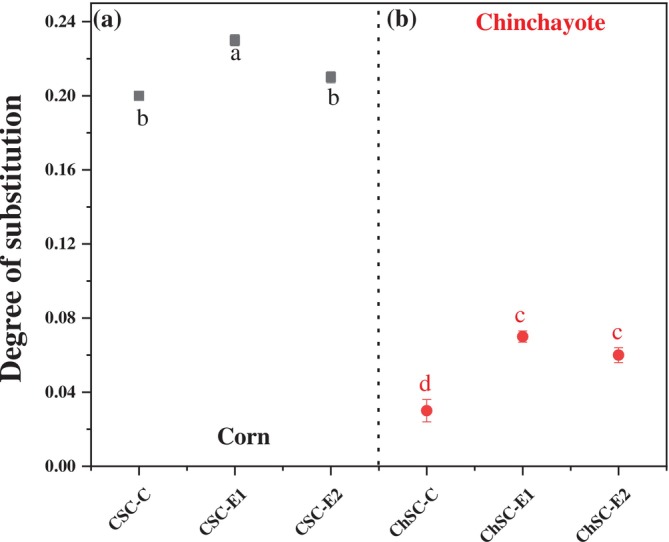
DS of cationized starches: (a) CSC‐C, CSC‐E1, and CSC‐E2, and (b) ChSC‐C, ChSC‐E1, and ChSC‐E2 with a concentration of 3% GTAC. Data are presented as mean ± standard deviation (*n* = 3). Different lowercase letters indicate statistically significant differences (*p* < 0.05).

The objective of the present study was to compare the efficiency of the conventional and extrusion methods, and to further compare the reaction of the cationizing (GTAC) with both starches. REX resulted in slightly higher degrees of substitution (DS) in CS (Figure 2a) compared to the conventional method, which can be attributed to shear‐induced structural modifications during extrusion. This process promoted the partial pregelatinization of the starch, enhancing the accessibility of hydroxyl groups within the polysaccharide chains and thus facilitating the incorporation and binding of cationic groups (GTAC) during the modification reaction. However, the DS for CSC‐E1 (0.23) was higher than that for CSC‐E2 (0.21), which can be attributed to a greater degree of gelatinization due to the temperature differences.

In other words, a higher degree of gelatinization leads to a decrease in the DS in cationic starch primarily due to structural changes in the starch granule during gelatinization. Native starch granules possess a semi‐crystalline structure composed of both amorphous and crystalline regions. During gelatinization‐induced by heat and moisture, these granules swell, lose their crystallinity, and undergo molecular disorganization, particularly involving the disruption of hydrogen bonds [32]. Moreover, the structural collapse increases the mobility and availability of hydroxyl groups, which suggests enhanced reactivity. However, in highly gelatinization starch, the molecular disarray and viscosity increase significantly, which affects the diffusion of the cationic reagent into the starch matrix [33, 34]. Moreover, once the granular integrity is lost, the reaction tends to become diffusion‐limited rather than chemically limited. Xu et al. [35] reported that the accessibility of internal hydroxyl groups is reduced due to gelatinization‐induced aggregation or retrogradation, thereby lowering the overall efficiency of the substitution reaction and, consequently, the final DS. Similar structural effects, including reduced crystallinity, granule aggregation, and increased gelatinization temperatures, have also been reported for chemically modified starches bearing aldehyde groups [36], indicating that the introduction of functional groups can significantly alter starch organization and thermal behavior.

In contrast, ChS exhibited lower DS values than corn starch under the same modification conditions (Figure 2b). This behavior can be attributed to its higher amylopectin content, which generates steric hindrance and limits the accessibility of hydroxyl groups for cationic substitution [37]. Additionally, the unique phosphorus content in tuber starches, as reported in chayote root starch by Morales‐Santiago et al. [19], may further impede the interaction between GTAC and starch chains due to electrostatic repulsion [38, 39]. However, the REX starches exhibited a higher average number of substituted hydroxyl groups than those treated through conventional cationization, suggesting that the extrusion process is more efficient for this type of modification.

### 3.3 Scanning Electron Microscopy (SEM)

**FIGURE 3:**
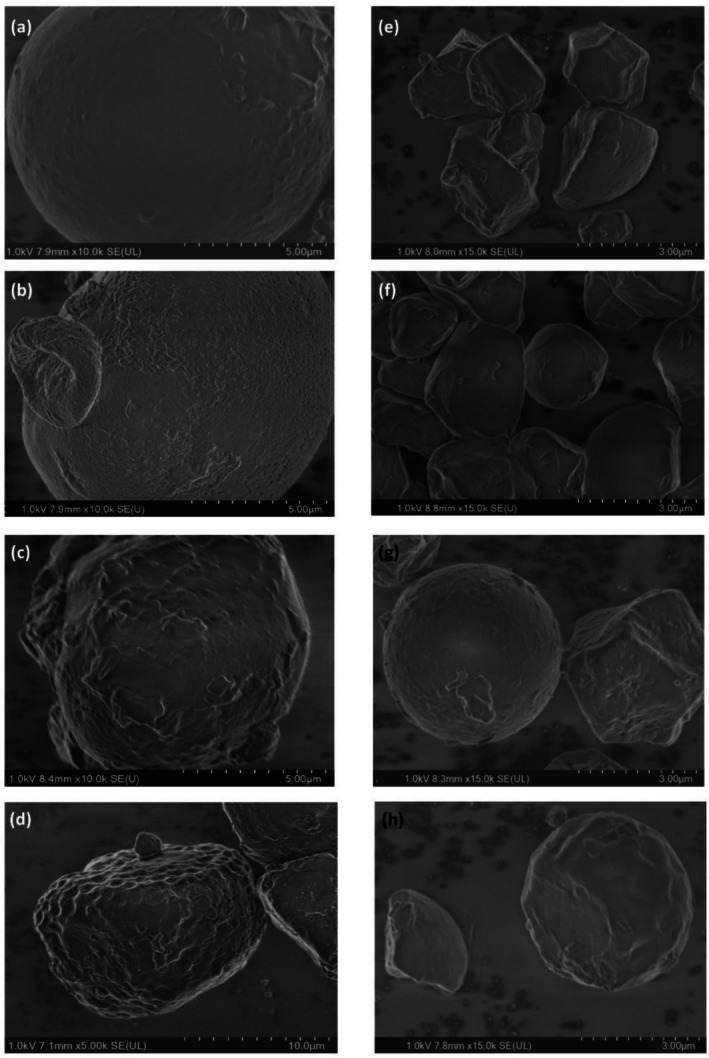
Microscopic images of native and modified starches: (a) CSN (10,000×), (b) CSC‐C (10,000×), (c) CSC‐E1 (10,000×), (d) CSC‐E2 (5000×), (e) ChSN (15,000×), (f) ChSC‐C (15,000×), (g) ChSC‐E1 (15,000×), and (h) ChSC‐E2 (15,000×).

Scanning electron microscopy (SEM) was utilized to examine the morphological changes in the starch granules after cationization through the conventional method and extrusion. CSN, where these granules exhibit different pointed edges with irregular, polygonal, or polyhedral shapes. As shown in Figure 3a, isolated CSN granules are smooth, showing no damage or fractures; the size distribution of isolated granules is 21.78 ± 5.66 μm, whereas conventional cationization caused only minor surface modifications, consistent with the relatively low degree of substitution. Similar behavior has been reported by El‐Naggar et al. [4], they observed minimal surface disruption in conventionally cationized corn starch, and by Nakasathien et al. [40], they reported that low degrees of substitution result in starch granules maintaining their integrity with only minor surface roughness. In contrast, starches modified by reactive extrusion showed more pronounced morphological alterations, including increased surface roughness and porosity (Figure 3b–d). These changes are attributed to the combined effects of shear forces and elevated temperatures during extrusion, which promote partial disruption of the granular structure [10]. Samples with higher degrees of substitution exhibited greater porosity, suggesting that processing severity plays a key role in structural modification.

In the case of ChS (Figure 3e), which features irregular shapes and good integrity, with a mean size distribution of 4.27 ± 1.14 μm. Conventional cationization resulted in minimal morphological changes, whereas reactive extrusion induced surface porosity while largely preserving granule integrity (Figure 3f–h). This behavior is consistent with observations reported for extruded starches and highlights the influence of botanical origin and processing conditions on starch morphology.

### 3.4 X‐Ray Structural Analysis

**FIGURE 4:**
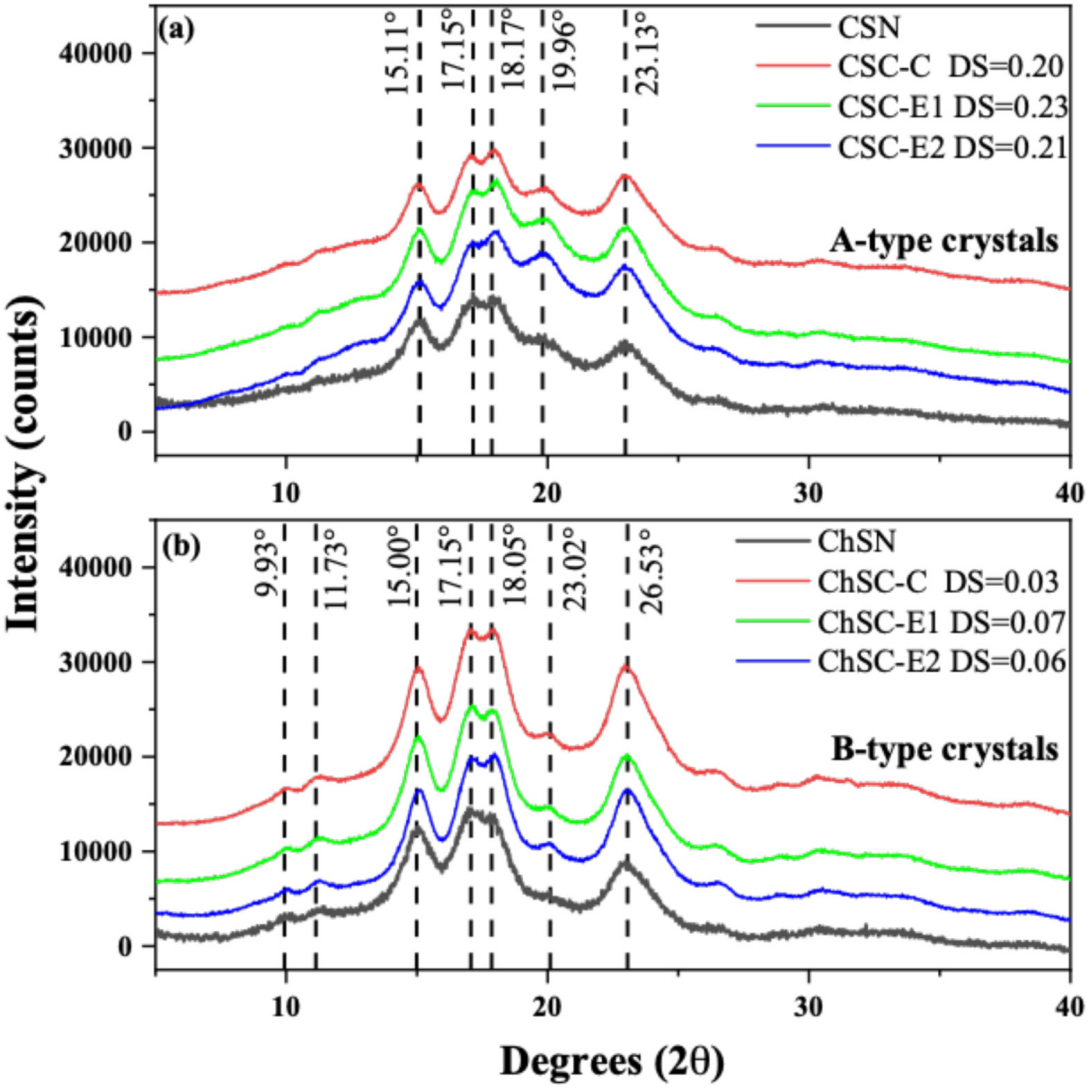
X‐ray diffraction patterns of native and modified starches: (a) corn starch (CSN, CSC‐C, CSC‐E1, and CSC‐E2) and (b) Chinchayote starch (ChSN, ChSC‐C, ChSC‐E1, and ChSC‐E2).

The diffraction patterns were analyzed to investigate the structural changes in starches produced by extrusion and conventional modification processes with the same GTAC concentration. Additionally, the extrusion process is influenced by several factors, including pressure, shear rate, humidity, and temperature, which can potentially lead to structural changes. As shown in Figure 4a, CSN has an orthorhombic structure, consistent with previous reports for Londoño‐Restrepo et al. [23], which is characteristic of amylopectin‐rich starches. After conventional modification, CSC‐C shows no discernible change in the diffraction pattern, attributable to a degree of substitution (DS) of 0.20. In contrast, the extruded starches CSC‐E1 and CSC‐E2 exhibit a reduction in crystallinity, as indicated by the peaks. However, the loss of crystallinity is more pronounced in CSC‐E1, due to the higher DS of 0.23. Furthermore, extrusion is a thermal and mechanical process that disrupts the original crystalline structure of the starch, particularly the ordered arrangement of the amylose and amylopectin chains [10].

In the case of ChSN (Figure 4b) exhibited XRD patterns consistent with a hexagonal crystalline structure, in agreement with previously reported data for isolated avocado pit starch and potato starch [22], which exhibits characteristic diffraction features indexed using the chiral space group *P6*_*1*_ [41]. After conventional modification, ChSC‐C exhibited behavior similar to that observed for CSC‐C, indicating that conventional cationization did not substantially alter the crystalline arrangement. In contrast, extruded chinchayote starches (ChSC‐E1 and ChSC‐E2) showed diffraction profiles comparable to those of extruded corn starches, characterized mainly by peak broadening and a reduction in relative crystallinity, rather than by the appearance of new crystalline reflections. Notably, the DS values of ChSC‐E1 and ChSC‐E2 (0.06 and 0.07, respectively) were lower than those of CSC‐E1 and CSC‐E2, which may be related to the amylopectin and phosphorus contents characteristic of starches with a hexagonal crystalline structure [37].

**FIGURE 5:**
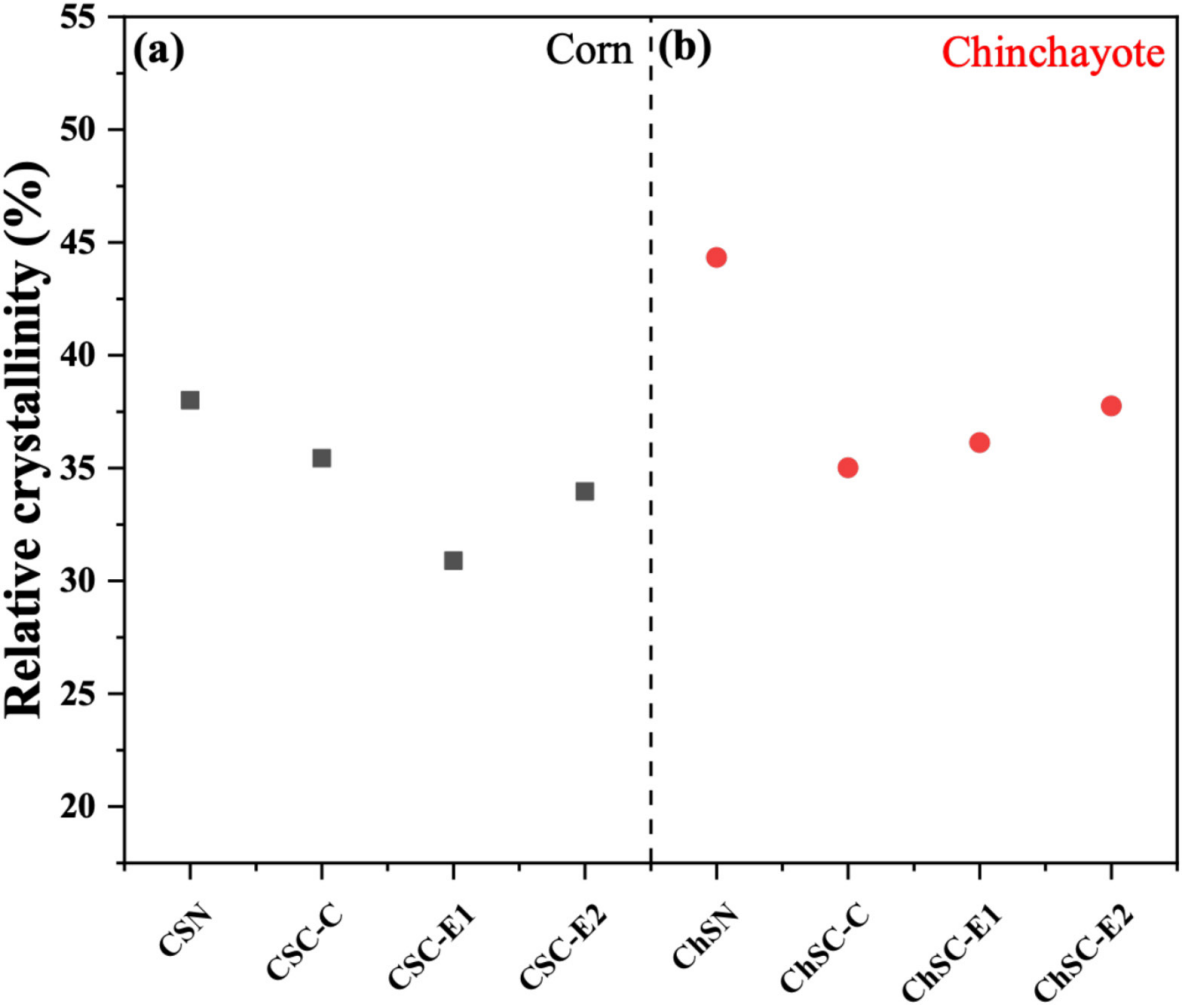
Relative crystallinity comparison of native and modified starches: (a) corn starch (CSN, CSC‐C, CSC‐E1, and CSC‐E2) and (b) Chinchayote starch (ChSN, ChSC‐C, ChSC‐E1, and ChSC‐E2).

Relative crystallinity (RC) of the native and modified starches for CS (Figure 5a) and ChS (Figure 5b) was calculated to evaluate the changes in starch crystallinity induced by CT and REX modifications. The decrease observed after conventional cationization is attributed to the introduction of cationic groups, which may affect molecular rearrangement during the cationization process and subsequent drying step. In contrast, the REX samples exhibited variations in relative crystallinity, remaining consistently lower than that of the native starch, which may be associated with amylopectin content, as previously reported for extruded corn starches [10].

For ChS, a greater reduction in relative crystallinity was observed after extrusion processing. Nevertheless, Almonaityte et al. [42] reported that the cationized potato starch presented changes in its crystalline structure associated with the degree of substitution. In this case, the high amylose content of chinchayote starch may promote molecular reorganization during cooling after extrusion, leading to partial reordering within the starch structure.

### 3.5 Differential (DSC)

In its natural state, starch exhibits various thermal transitions, such as gelatinization, which reflect the interaction and organization of the amylose and amylopectin molecules within its structure [43]. The thermal transitions of cationized starch modified by conventional and extrusion processes were analyzed using DSC, as it provides a comprehensive representation of the modified starch structural and thermal changes.

**FIGURE 6:**
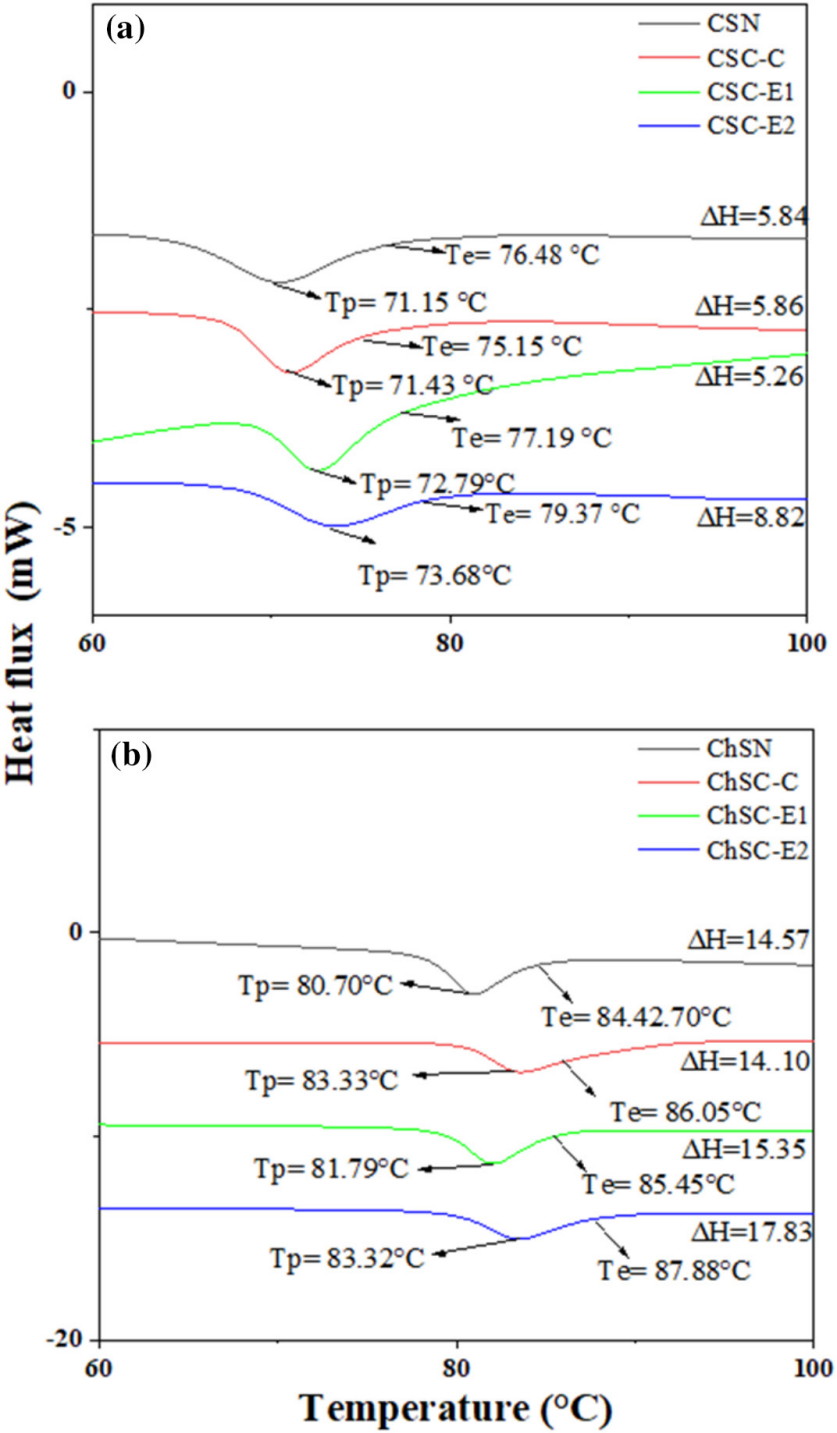
DSC thermal analysis of native and modified starches: (a) corn starch (CNS, CSC‐C, CSC‐E1, and CSC‐E2) and (b) Chinchayote starch (ChSN, ChSC‐C, ChSC‐E1, and ChSC‐E2).

Table 2 presents the onset (*T*_o_), peak (*T*_p_), and final (*T*_e_), corresponding to the completion temperature (*T*c), along with the enthalpy (∆H) of the phase transition for both native and modified starches. For CS (Figure 6a), cationization by conventional and extrusion processes resulted in noticeable changes in the gelatinization behavior compared to the native sample. The extrusion process facilitates the penetration of the cationizing agent into the starch structure through the combined effects of shear and temperature, promoting partial pregelatinization and enhancing the efficiency of the modification, which is reflected in the thermal behavior observed by DSC. In general, cationized samples exhibited higher transition temperatures than native starch, particularly those modified by extrusion.

**TABLE 2:** Thermal properties and pasting profiles of native and modified starches: (a) corn starch (CSN, CSC‐C, CSC‐E1, and CSC‐E2) and (b) Chinchayote starch (ChSN, ChSC‐C, ChSC‐E1, and ChSC‐E2).

Sample	*T*_o_ (°C)	*T*_p_ (°C)	*T*_e_ (°C)	∆*H* (J/g)	Pasting temperature (°C)	Peak viscosity (cP)	Final viscosity
CSN	64.47	70.17	76.48	8.82 ± 0.25^c^	49.07 ± 0.86^b^	5854.96 ± 34.67^ab^	4214.31 ± 45.74^ab^
CSC‐C	67.59	70.85	75.15	5.86 ± 0.20^a^	48.88 ± 0.46^b^	7366.64 ± 89.17^a^	4214.31 ± 57.91^a^
CSC‐E1	69.11	72.57	77.19	7.26 ± 0.42^b^	48.09 ± 0.57^b^	4178.41 ± 846.10^cd^	2603.93 ± 80.55^cd^
CSC‐E2	68.40	73.36	79.37	5.84 ± 0.71^a^	48.14 ± 0.06^b^	2647.36 ± 693.29^d^	2090.28 ± 40.91^d^
ChSN	77.96	80.70	84.42	17.83 ± 0.71^e^	69.22 ± 0.66^a^	6160.30 ± 52.97^ab^	3162.91 ± 78.88^bcd^
ChSC‐C	81.38	83.33	86.05	14.57 ± 0.71^d^	42.59 ± 1.52^b^	4654.79 ± 301.02^bc^	3348.54 ± 502.36^bcd^
ChSC‐E1	79.05	81.79	85.45	15.35 ± 1.41^d^	49.40 ± 5.11^b^	5886.76 ± 488.33^abc^	3723.29 ± 200.07^abc^
ChSC‐E2	79.87	83.32	87.88	14.10 ± 1.41^d^	28.79 ± 0.32^c^	5049.19 ± 320.74^bc^	3577.57 ± 826.40^abc^

*Note:* Data are presented as mean ± standard deviation (*n* = 3). Different lowercase letters indicate statistically significant differences (*p* < 0.05).

These changes may be associated with the degree of substitution (DS), since samples with higher DS showed increased gelatinization temperatures, suggesting enhanced thermal stability. According to Utrilla‐Coello et al. [44] reported that during the cationization process, the introduction of cationic groups into the starch weakens the structure due to the repulsion between adjacent groups, which inhibits the associations between chains and promotes increased interactions with water molecules. Consequently, a decrease in gelatinization enthalpy was observed in modified samples, especially those processed by extrusion, which can be attributed to structural disorganization and partial pregelatinization occurring under high temperature and shear conditions. This behavior has been related to the degree of substitution achieved during the modification process, resulting in lower energy requirements for gelatinization [45].

A similar trend was observed for ChS (Figure 6b), where both conventional and extrusion cationization led to slightly higher gelatinization temperatures and a reduction in enthalpy compared to the native starch, as summarized in Table 1. These results indicate that the modification processes induce structural rearrangements that affect the thermal stability and energy demand of gelatinization.

As shown in Table 2, the conventional cationization procedure resulted in increased gelatinization of the ChSC‐C sample. This outcome can be attributed to the extended exposure of the starch to the reagents and process conditions, including temperature and constant stirring during the reaction [46, 47].

### 3.6 Viscosity Profile

**FIGURE 7:**
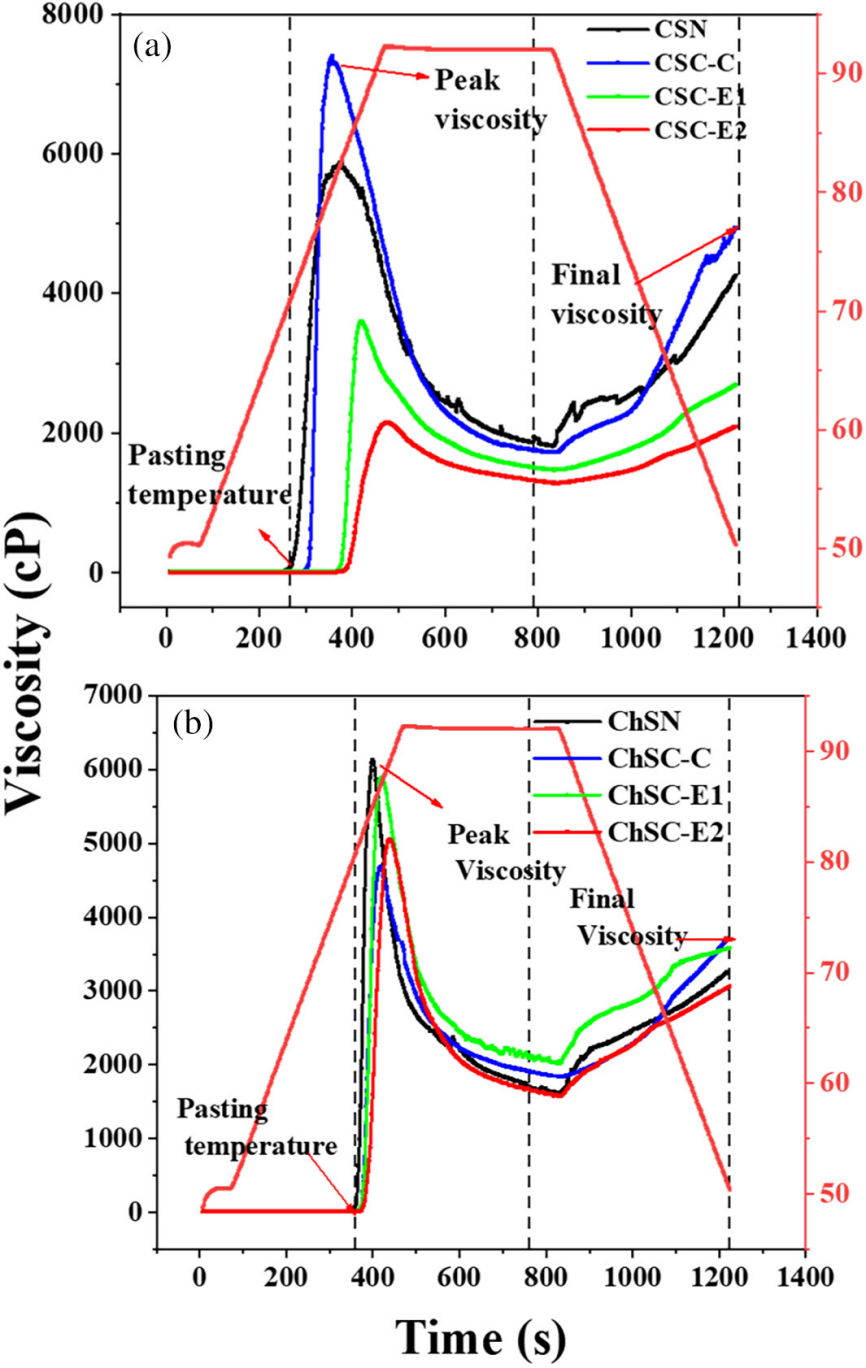
RVA analysis of native and modified starches: (a) corn starch (CSN, CSC‐C, CSC‐E1, and CSC‐E2) and (b) Chinchayote starch (ChSN, ChSC‐C, ChSC‐E1, and ChSC‐E2).

Rapid Visco Analyzer (RVA) analysis (Figures 7a and 7b) allows for real‐time measurement of viscosity changes during the heating and cooling processes, offering a comprehensive profile of gelation, peak viscosity, heat, and shear stability [48]. The change in viscosity of native and modified corn and chinchayote starches through conventional and extrusion methods was examined using variable‐angle rheometry (RVA). The results indicate that CT starch showed higher peak viscosity than native starch (Table 2), while REX samples exhibited lower viscosity due to pre‐gelatinization. This behavior is associated with partial pre‐gelatinization and structural disruption induced by shear and temperature during extrusion, which limits granule swelling and reduces paste viscosity. Variations in viscosity parameters were closely related to the degree of substitution (DS), with higher DS values generally corresponding to lower peak and final viscosities.

In the case of CSC (DS = 0.20), the DS was lower compared to CSC‐E1 (DS = 0.23) and CSC‐E2 (DS = 0.21) (Figure 7a). This was reflected in the temperature, which decreased from 86°C to 80°C. Notably, the viscosity of the modified starches by extrusion is lower. This can be attributed to the preceding gelatinization and depolymerization processes due to shearing. In the case of CSC‐E1 and CSC‐E2, a difference in the paste temperature time of the native starch compared to the extrusion‐modified starch can be observed, which is attributed to the shearing process and moisture content. The final viscosity peaks of the extruded starches show a decrease due to the damage of the granular structure, consistent with the morphological changes observed by SEM.

A similar trend was observed for ChSN (Figure 7b). Modified samples showed lower peak viscosity than the native starch, and higher degrees of substitution were associated with greater viscosity reduction. The limited changes observed in paste temperature and final viscosity among extruded chinchayote starches may be related to their botanical origin and intrinsic molecular organization. Comparable effects of extrusion on starch pasting behavior have been previously reported and attributed mainly to the thermomechanical conditions of the extrusion process, such as heat treatment, mechanical work, and moisture, rather than to specific chemical modification pathways [10].

### 3.7 FTIR Analysis

FTIR has been utilized to study the modification of cationized starch using both conventional methods and extrusion, as this technique can detect functional groups [49]. The spectra of native and modified starches revealed that the overall fingerprint region remained largely unchanged after modification, indicating that the main polysaccharide backbone was preserved.

The appearance of the band around 1639 cm^−1^, assigned to C—N stretching vibrations, confirms the successful incorporation of cationic groups into the starch structure [50]. The cationization process of α‐glucose into positively charged functional groups is attributed to incorporating C and N atoms into the reticulum, demonstrating a physicochemical change.

**FIGURE 8:**
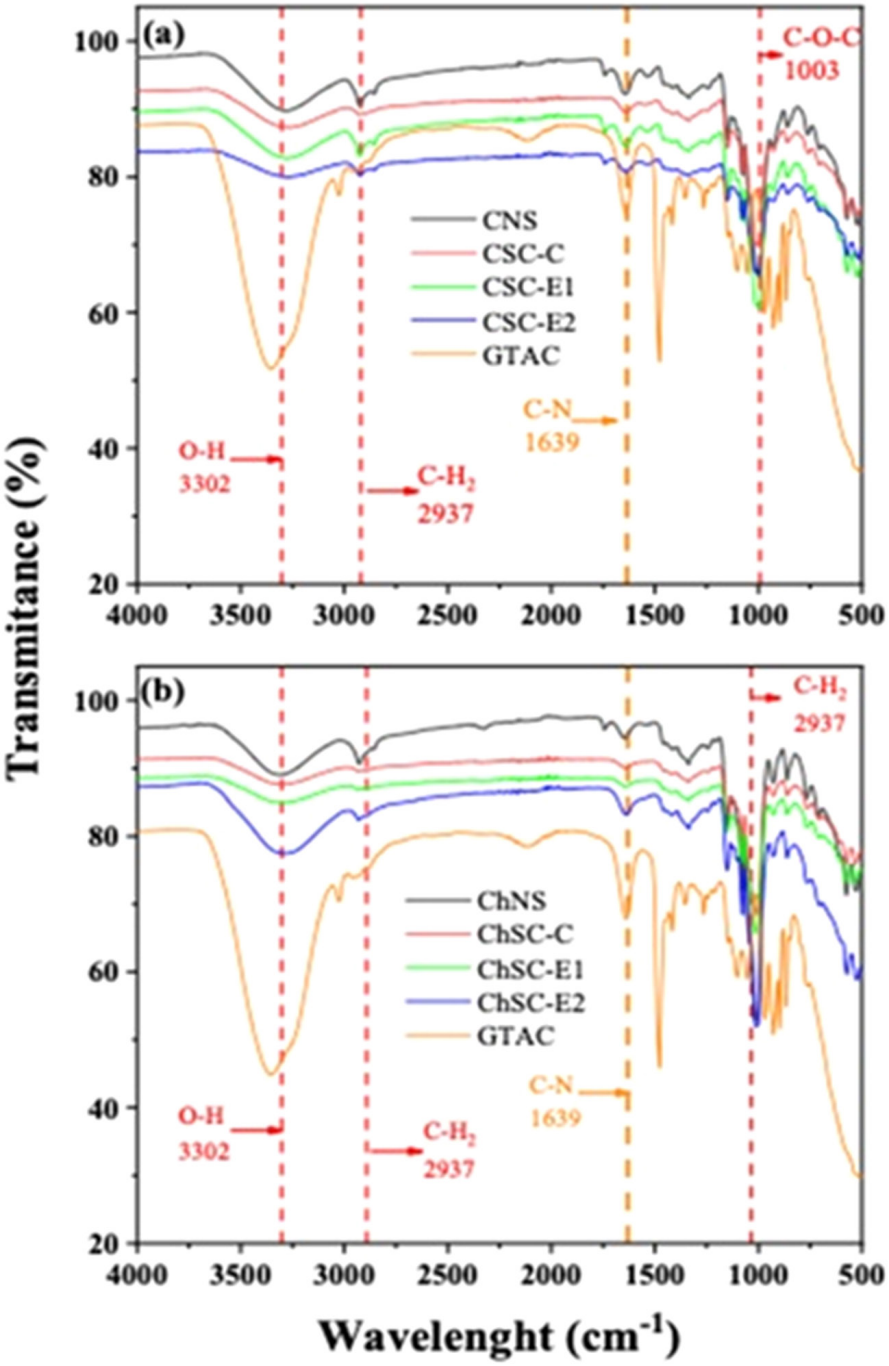
FTIR analysis of native and modified starches: (a) corn starch (CSN, CSC‐C, CSC‐E1, and CSC‐E2), (b) Chinchayote starch (ChSN, ChSC‐C, ChSC‐E1, and ChSC‐E2), and GTAC (orange line).

Variations in band intensity, particularly in the O—H stretching region, suggest alterations in hydrogen bonding and molecular order following cationization and extrusion. These changes are associated with partial disruption of the ordered starch structure and an expansion of the amorphous regions due to the presence of GTAC, as shown in Figure 8a,b. In extrusion‐modified samples, additional intensity variations were observed, which can be attributed to the combined effects of temperature, shear, gelatinization, and subsequent molecular rearrangement during cooling.

Similar spectral trends were observed for both corn and chinchayote starches, indicating that the modification mechanisms induced by conventional cationization and reactive extrusion are comparable regardless of botanical origin. The characteristic FTIR bands discussed above are summarized in Table 3.

**TABLE 3:** Band position of native and modified starches.

Band assignment	Band position (cm^−1^)								References
	Corn				Chinchayote				
	CSN	CSC‐C	CSC‐E1	CSC‐E2	ChSN	ChSC‐C	ChSC‐E1	ChSC‐E2	
O—H stREXching	3302	3292	3279	3301	3302	3275	3263	3267	Bustillos‐Rodríguez et al. [51]
C—H_2_ antisymmetric stREXch	2937	2926	2919	2921	2937	2943	2946	2980	Sumayya et al. [52]
C—N stREXch	—	1639	1642	1638	—	1637	1643	1643	El‐Naggar et al. [4]
C—O—C glycosidic linkage	1138	1140	1142	1145	1136	1142	1144	1146	Awolu et al. [50]
C—O—H stREXch	1002	1004	1101	1005	1010	1012	1013	1014	Hong et al. [53]

## 4 Conclusion

Corn and chinchayote starches were successfully cationized using both conventional treatment (CT) and reactive extrusion (REX) with GTAC as the cationizing agent. The comparative analysis demonstrated that REX is a more efficient modification strategy, as it significantly reduced processing time and reagent consumption while effectively incorporating cationic groups into the starch structure.

Structural, thermal, and rheological characterizations confirmed that reactive extrusion induces more pronounced physicochemical changes than the conventional method, mainly due to the combined effects of shear, temperature, and mechanical energy during processing. These changes were dependent on the botanical origin of the starch, highlighting the influence of intrinsic structural features on modification efficiency.

Overall, REX was identified as a viable and sustainable alternative to conventional cationization, offering advantages in terms of energy efficiency, reduced solvent use, and industrial scalability. The results support the potential application of REX‐modified starches in value‐added uses such as water treatment, flocculation processes, and the development of environmentally friendly materials. Further studies should focus on process optimization and validation at an industrial scale.

## Author Contributions

**Maria C. Posada‐Vélez:** investigation, validation. **Beatriz M. Millán‐Malo:** investigation, conceptualization, writing, formal analysis. **Oscar Y. Barrón‐García:** supervision, formal analysis, writing – review and editing. **Marcela Gaytán‐Martínez:** conceptualization, methodology, formal analysis, writing – original draft, supervision, writing – review and editing. **Germán Buitrón:** investigation, formal analysis.

## Funding

The work was supported by Laboratorio Nacional de Caracterización de Materiales (LaNCaM)‐SECIHTI at the Centro de Física Aplicada y Tecnología Avanzada (CFATA), Universidad Nacional Autónoma de Mexico (UNAM); the Centro de Investigación en Ciencias Aplicadas y Tecnología Avanzada (CICATA) of the Instituto Politécnico Nacional (IPN).

## Conflicts of Interest

The authors declare no conflicts of interest.

## Data Availability

The data that support the findings of this study are available from the corresponding author upon reasonable request.
